# Neurotherapeutics across blood–brain barrier: screening of BBB-permeable and CNS-active molecules for neurodegenerative disease

**DOI:** 10.3389/fphar.2025.1616144

**Published:** 2025-09-26

**Authors:** D. Mohan Kumar, Priti Talwar

**Affiliations:** Apoptosis and Cell Survival Research Laboratory, 412G Pearl Research Park, Department of Biosciences, School of Biosciences and Technology, Vellore Institute of Technology, Vellore, Tamil Nadu, India

**Keywords:** neurotherapeutics, BBB permeable, CNS active, small molecules, drug discovery, pharmacokinetics, neurotrophic factor

## Abstract

Neurotherapeutics that are effective in the central nervous system (CNS) of the brain require an accurate estimation of their uptake across the blood–brain barrier (BBB), a highly selective membrane between the bloodstream and the nervous system that restricts and regulates the entry of small molecules. Drugs that influence the CNS must permeate the BBB prior to reaching their target site. Therefore, the prediction of BBB permeability with CNS activity is a fundamental aim and significant research objective in neuropharmacology. Here, we utilized *in silico* approaches and available machine learning models ranging from physicochemical properties to structure–activity relationships in a CNS drug discovery pipeline to identify BBB-permeable molecules. These models pertain to pharmacophore-based virtual screening, BBB permeability and CNS activity prediction, medicinal chemistry, ADME, toxicity profiling, drug-likeness, side effect resources, and bioactivity studies. A total of 2,127 active small molecules were initially screened based on the structure similarity of five FDA-approved drugs of particular interest for neurodegenerative diseases. Based on the BBB model, they were classified into 582 BBB permeable and 1545 BBB non-permeable molecules. Most of the BBB-permeable molecules were reported to have direct CNS activity due to their high brain-to-blood ratio. Finally, 112 active CNS molecules were prioritized based on pharmacokinetics, toxicophores, and drug-likeness. Additionally, the neuroactivity toward the CNS of small molecules was predicted to be a nootropic, neurotrophic factor enhancer, and neuroinflammatory modulator. Thus, by ensuring their impact on BBB integrity and the neuroprotective properties of small molecules, they can in future be transformed into food supplements and nutraceuticals that could provide valuable insights into neurotherapeutics as promising therapeutic interventions for neurodegenerative diseases.

## 1 Introduction

Central nervous system (CNS) diseases comprise a wide range of medical conditions that affect the spinal cord and brain. Neurodegenerative diseases (NDDs) represent a significant subset of CNS disorders characterized by the progressive degeneration of nerve cells. NDDs are age-associated multifactorial diseases characterized by dementia, cognitive impairment, memory decline, motor dysfunction, progressive loss of neuronal cells, and extensive brain damage (Baswar et al., 2021). Aging is recognized as a primary risk factor for the progression of neurodegeneration, which results in significant impairment in human wellbeing and health (Duc Nguyen, 2023). The incidence and prevalence of age-related neurodegeneration, which are predominantly observed in clinical practice, primarily encompass Alzheimer’s disease, Parkinson’s disease, and amyotrophic lateral sclerosis and are increasingly recognized as major causes of death and disability worldwide. The pathophysiological hallmarks among these NDDs are misfolded protein accumulation, oxidative stress, neuroinflammation, and mitochondrial dysfunction (Wang et al., 2010). The aging population around the world is contributing to the rise in the occurrence of neurological diseases, constituting a significant threat to healthcare systems. To address this problem, there is an urgent need to discover and develop novel neurotherapeutic strategies that can effectively target the underlying mechanisms of these neurological diseases (Duc Nguyen, 2023; Wang et al., 2010).

Neurotherapeutics represents a multidisciplinary field in neuroscience that focuses on developing novel treatments for neurological disorders. It comprises various applications such as therapeutic interventions, pharmacological agents, gene therapies, cognitive behavioral therapies, and neuromodulation techniques. These therapies seek to alleviate symptoms, prevent disease progression, and ultimately improve patient outcomes in various neurological conditions (Smith et al., 2012). Neurotherapeutic drug development aims to identify new treatments that will increase the quality of life of individuals suffering from neurologic disorders to NDDs. Progress in neurotherapeutics and neuropharmacology with respect to clinical methodology employing therapeutic agents was used to successfully treat both primary neurologic illnesses and neurodegenerative conditions (Cummings, 2006). Neurotherapeutic approaches have been utilized in NDD treatments of the CNS, resulting in the creation of biologically active molecules as medicines that particularly target underlying mechanisms involved in the disease etiology. However, the identification of active molecules has faced some difficulty in treating neurodegenerative conditions due to the presence of a specialized microvascular unit, known as the “blood–brain barrier” (BBB), that is crucial for the drug development process (Lawal et al., 2018).

The BBB is the complex network of brain microvessels that separates the CNS compartment from peripheral blood circulation. The primary cells that make up the BBB are brain microvascular endothelial cells, which are held together by neuronal cells such as astrocytes and pericytes that act according to the state of the CNS. Hence, the BBB plays a vital role in protecting the CNS by regulating the stability of the physiological (internal) environment of brain tissue, maintaining brain homeostasis and preserving neuronal viability (Abbott et al., 2010). The BBB facilitates the supply of essential nutrients necessary for the normal functioning of the brain. The highly selective nature of the BBB allows only specific molecules to pass through it and enter the brain, as it has the least permeable capillaries due to physical barriers (tight junctions). This has been considered as a major obstacle in designing and delivering beneficial drug-like compounds into the brain via the BBB to treat CNS diseases (Geldenhuys et al., 2015). However, the restrictive nature of the BBB allows only 2% of biologically active small molecules to cross the intact BBB to reach the brain at varying degrees (Mikitsh and Chacko, 2014). For many neurological diseases, there is almost no effective treatment due to the insufficient permeability of therapeutic agents into the brain through the BBB. Therefore, screening small molecules as to their BBB permeability is a prerequisite for a drug discovery process for treating CNS disorders and NDDs (Nagpal et al., 2013).

Neurological disorders necessitate prolonged and lifelong therapeutic interventions. Current therapeutic approaches predominantly rely on synthetic drugs that are commonly used to treat most NDDs which have adverse reactions and side effects (Feng et al., 2023). Natural products and dietary-based molecules have exhibited significant therapeutic efficacy in preventing major diseases. Additionally, natural resources provide a unique way for the identification of promising novel chemicals with validated efficacy. It is estimated that nearly 50% of all newly approved drugs can be traced back to a structural origin derived from a natural product (Zhang et al., 2017). There is a growing interest in exploring active molecules from diet or dietary interventions and functional foods that possess neuroprotective properties that may enhance their wellbeing and potentially slow down the progression of neurodegeneration. These approaches may provide neuroprotective benefits with fewer side effects than conventional pharmacological treatments. As research continues to evolve in this field, the integration of dietary interventions as complementary strategies with innovative drug-delivery approaches may facilitate the effective management of NDDs (Feng et al., 2023).

The development of neurotherapeutics is significantly impacted by accurate predictions of BBB permeability and CNS activity. Existing traditional methods of assessing BBB permeability are often very challenging, time-consuming, require large-scale experimental trials, and are laborious, resulting in low throughput (Shaker et al., 2023) and are hence unsuitable for screening large libraries of molecules. Therefore, given these experimental difficulties, there is a pressing need for innovative approaches that can facilitate the rapid screening of drug-like candidates for their ability to permeate the BBB (Könczöl et al., 2013). Recent advances in computational-aided drug development (CADD) have emerged as powerful techniques in drug discovery, offering the potential to screen active molecules and predict BBB permeability based on physicochemical and pharmacokinetic properties with higher predictability and clinical applicability (Aldewachi et al., 2021). Therefore, multiple computational tools, *in silico* techniques, and existing machine learning model-based approaches have been introduced to screen large libraries of compounds or small molecules that can quickly predict the BBB permeability of active compounds. Such predictive models are highly beneficial in enhancing the early phase of the drug discovery process, especially in the field of CNS research (Stéen et al., 2022). Accelerating the drug discovery process is essential for performing high-throughput virtual screening of drug-like small molecules to evaluate the BBB permeability of the active molecules.

**FIGURE 1:**
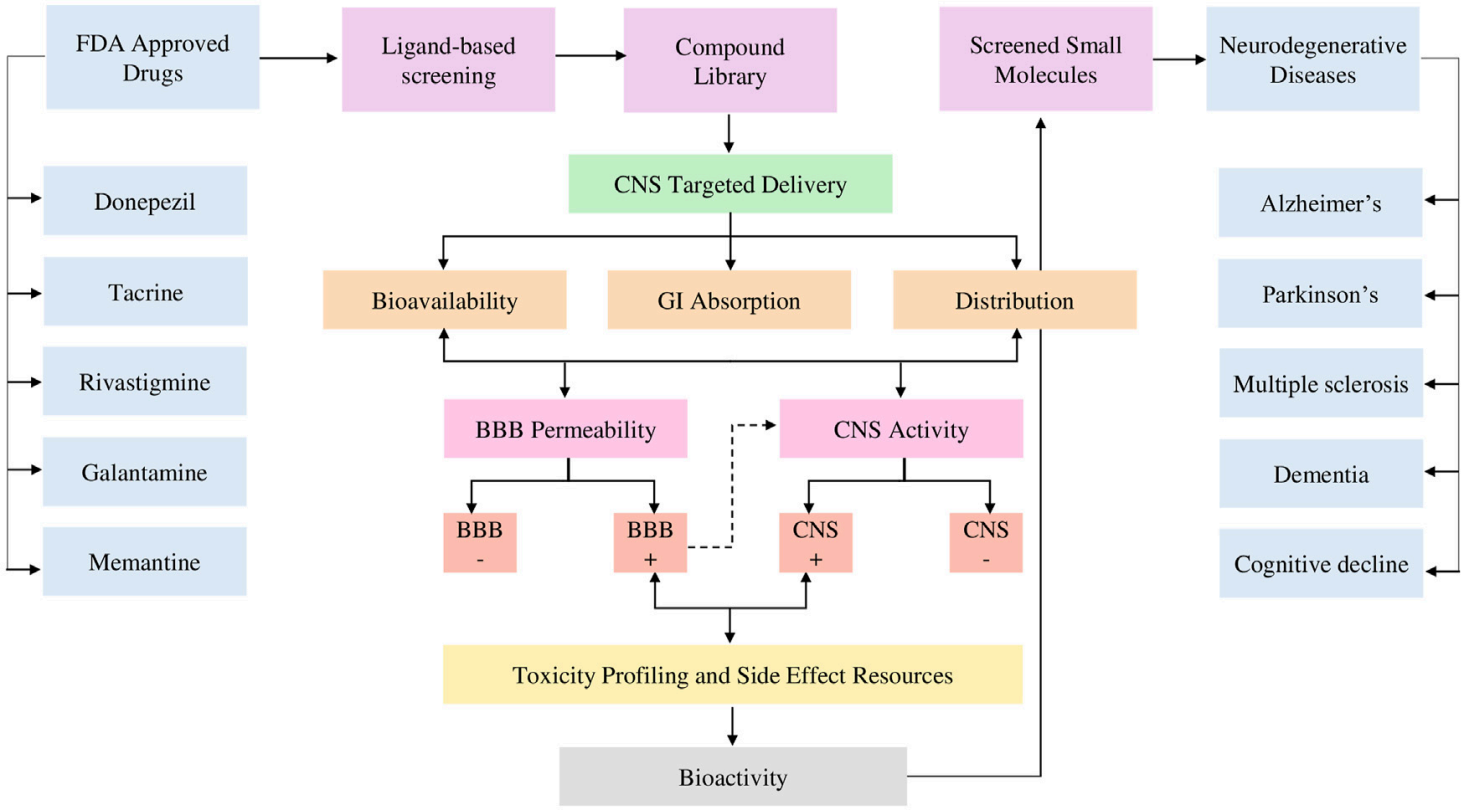
Research workflow starts from ligand-based screening based on a pharmacophore model of five FDA-approved drugs and compound library development. The process emphasizes CNS-targeted delivery, focusing on key pharmacokinetic factors such as bioavailability, gastrointestinal (GI) absorption, and distribution further evaluated for BBB permeability and CNS activity. Toxicity profiling and side effect resources are integrated before assessing bioactivity. The ultimate goal is to screen molecules for the treatment of NDDs.

This research applied *in silico* techniques together with machine learning and deep learning-based approaches in order to screen and predict the BBB permeability of druglike biologically active molecules from natural products and dietary sources. We explored the BBB permeability of CNS-active small molecules by interpreting physicochemical descriptors to compute the brain-to-blood ratio, as these metrics are routinely utilized to predict clinical exposure in CNS during the process of drug discovery (Zhang et al., 2016). Hence, we started with ligand-based virtual screening based on structural similarity to FDA-approved drugs. Then, the active molecules were screened and filtered based on multiple parameters, including BBB permeability, CNS activity, ADME profiling, toxicity, drug-likeness, bioavailability, medicinal chemistry, side effect resources, and bioactivity studies. Figure 1 provides a research workflow for screening BBB-permeable CNS-active molecules. All molecules were evaluated for the following parameters in order to identify drug-like small molecules: improved pharmacokinetics, pharmacodynamics, physicochemical properties, and the ability to directly activate at the CNS level by BBB permeation. Therefore, the incorporation of computational approaches in screening CNS-active and BBB-permeable molecules in the early phase of the drug discovery process can reduce the later-stage attrition rate and improve the overall success rate of drug discovery and development. This may enable the discovery of small molecules from natural sources with appropriate BBB permeation to elicit their bioactivity response against neurotherapeutic targets (Dichiara et al., 2024).

## 2 Materials and methods

### 2.1 Pharmacophore-based virtual screening

Pharmit (Sunseri and Koes, 2016), ChemMine (Backman et al., 2011), and Swiss similarity (Zoete et al., 2016) were used for ligand-based pharmacophore modeling and screening of molecules. The five FDA-approved drugs as a pharmacophore model were selected, and they were imported as a query molecule in the Pharmit server, ChemMine, and Swiss similarity to screen large libraries of structurally similar molecules. Virtual screening was based on similarity value, calculated according to the Tanimoto similarity score. Pharmit, ChemMine, and the Swiss similarity web servers are a collection of inbuilt databases such as PubChem, Drugbank, Zinc15, Chemspace, ChEMBL, CHEBI, Molprot, and ZINCPharmer used to screen structurally similar molecules (Sunseri and Koes, 2016; Backman et al., 2011; Zoete et al., 2016). NPClassifier, a deep-learning tool for structurally classifying natural products, was utilized to classify the molecules (Kim et al., 2021).

### 2.2 Computing molecular descriptors

Molecular descriptors related to BBB permeability and CNS activity were computed by an integrated web-based platform ChemDes (ChemoPy Descriptor Calculator) (Dong et al., 2015). ChemDes can compute all descriptors that can be calculated by utilizing Python modules of ChemoPy, chemistry development kit, RDKit, and PaDel descriptors in order to represent each active compound (Yuan et al., 2018). The SMILES strings of bioactive molecules or compounds were firstly uploaded to ChemDes to calculate 3D molecular descriptors like geometric descriptors, topological descriptors, physicochemical descriptors, charged partial surface area descriptors, and the molecular representation of structure-based descriptors.

### 2.3 Predicting BBB permeability

BBB permeability was evaluated to compute the potential of active molecules to permeate the BBB using machine learning-based quantitative models like BBBper (Kumar P. et al., 2024), LightBBB (Shaker et al., 2021), and online BBB predictor (Liu et al., 2014), which are based on several machine learning algorithms such as support vector machine (SVM), random forest (RF), AdaBoost, and XGBoost to predict the BBB permeability of bioactive molecules. Deep-B3, a deep-learning-based model, was used to evaluate the BBB permeability of molecules (Tang et al., 2022). These models aid in the early phase of the high-throughput screening of BBB-permeable molecules and also improve the success rate in the development of CNS drugs. Each molecule structure (as SMILES format) is used as input for multiple online platforms like BBBper, LightBBB, and Deep-B3 to list whether the molecule is BBB-permeable (BBB+) or -non-permeable (BBB−).

### 2.4 CNS activity

The LogBB_Pred web server was utilized to compute the CNS activity of BBB-permeable compounds; it accepts a list of bioactive molecules in SMILES strings and in return provides predicted logBB values (Shaker et al., 2023). Consequently, this model can accurately classify the CNS activity of BBB permeable compounds based on the absolute log [Brain]/[Blood] (logBB) values (i.e., the ratio in the brain to that in the blood) of drug-like molecules. Small molecules were considered to be CNS-active if their logBB value exceeded a defined threshold value (usually ≥ −1) to evaluate the activity of the compound at the CNS level (Shaker et al., 2023). Additionally, the CNS multiparameter optimization (MPO) algorithm was used to calculate CNS MPO scores (desirability score values ≥4.0), combining physicochemical and pharmacokinetic properties to assess CNS-active druglike molecules (Wager et al., 2016).

### 2.5 Medicinal chemistry metrics

The Swiss-ADME web tool was employed to evaluate the medicinal chemistry friendliness of bioactive molecules (Daina et al., 2017). The following filters were applied for further screening: pan-assay interference compounds (PAINS), Brenk alerts, lead-likeness, and synthetic accessibility (Opo et al., 2021). The PAINS substructure filter rule was proposed to exclude promiscuous compounds that may interfere with assays. Brenk structural alerts detect reactive functional groups. Lead-likeness was applied to determine whether a molecular entity is suitable for optimization. The synthetic accessibility score estimates the ease of synthesizing the compounds in the laboratory. Medicinal chemistry rules were applied with the standard settings already implemented in the FAF-Drugs4, which have become an essential component in early-stage drug discovery (Stork et al., 2021).

### 2.6 ADME profiling

The ADMET lab 2.0 (Xiong et al., 2021), Swiss ADME (Daina et al., 2017), and the pkCSM ADME (Pi et al., 2015) online servers were widely used for the systematic evaluation of the pharmacokinetic properties of screened compounds relative to CNS drug suitability, provided with diverse physicochemical associations with known absorption, distribution, metabolism, and excretion. ADME screening is a batch mode for evaluation, designed for the prediction of pharmacokinetics based on the molecular submission of supported SMILES strings and structural data formatted files. ADMET lab 2.0 (Xiong et al., 2021) and Swiss ADME (Daina et al., 2017) were utilized to evaluate intestinal absorption (human) through Caco-2 and MDCK permeability in terms of absorption. The volume of distribution (Vd), BBB permeability, and CNS activity were evaluated in terms of distribution. Furthermore, cytochrome P450 isoforms substrate or inhibitor were examined with respect to metabolism. In addition, the pkCSM (Pi et al., 2015) online server was accessed to predict Renal OCT2 substrate or inhibitor and total clearance of compounds through excretion.

### 2.7 Bioavailability

The HobPre (human oral bioavailability prediction resource) *in silico* method was utilized to accurately predict the human oral bioavailability of small molecules. We began by inputting the SMILES representations of the compounds into the HobPre web application, which utilized a consensus model based on five random forest classifiers to predict human oral bioavailability (HOB). The model integrated various approaches like machine learning, pharmacokinetic modeling, and molecular descriptors to predict oral bioavailability from chemical structure and to classify the molecules based on predictive bioavailability threshold values (Wei et al., 2022). Additionally, Swiss ADME was employed to find the oral bioavailability based on the bioavailability radar model of drug-like small molecules.

### 2.8 Estimation of toxicity

ProTox 3.0 (Banerjee et al., 2024) was used in this study to estimate the toxic effects of each molecule. ProTox, an advanced virtual toxicity lab, was used for the prediction of multiple toxicological factors related to molecular structure, pharmacophore mapping, and fragment-based propensity scoring to predict a comprehensive range of toxicological endpoints. It was used to calculate acute oral toxicity (expressed as LD50, mol/kg) and predict toxicity classes, ranging from Classes 1 (extremely toxic) to 6 (non-toxic) for each input compound based on chemical similarities to toxic compounds and a set of trained machine-learning models. ProTox-3 was then used to determine organ-specific toxicity, other toxicity endpoints, and Tox21-toxicological pathways (Banerjee et al., 2024).

### 2.9 Evaluation of drug-likeness

The drug-likeness of bioactive compounds was estimated by cheminformatics techniques with the aid of computational tools such as Drulito (Bickerton et al., 2012) and Molsoft (Molsoft, 2025). The Drulito tool was utilized to identify drug-likeness properties based on Lipinski’s “rule of five” (RO5) and other sets of basic rules such as the Ghose filter, Veber rule, Egan rule, and Muegge rules at definite threshold value to determine the drugability of each molecule. These rules are useful in screening and discovering drug-like molecules based on structure–activity (Ani et al., 2020). Molsoft was assessed to select drug-like candidates based on the drug-likeness score. Drug-like soft filter was executed based on several physicochemical parameters of drugs integrated with an in-built statistical analysis of approved drugs (Tamaian et al., 2023).

### 2.10 SIDER-side effects resource

The SIDER online database was utilized; it contains information on the recorded side effects (details about adverse drug reactions) extracted from public repositories (Kuhn et al., 2016). The drug-like molecules were searched against the SIDER database with their PubChem CID. Currently accessible information includes associated side effects, frequency of side effects, side effect classification (e.g., frequent, infrequent, and rare), and connections to further resources, such as drug–target relations. The drug clinical phenotypes in the SIDER 4.1 dataset were implemented according to MedDRA. The phenotypes in MedDRA are organized in a five-level hierarchical structure and were employed to extract a specific clinical phenotype for mapping symptoms, signs, diagnoses, and therapeutic indications under CNS and neurological disorders (Kuhn et al., 2016; Gao et al., 2017).

### 2.11 Prediction of bioactivity and mechanism of action

Way2Drug-PASS online and Molinspiration web tools were used to explore the bioactivity of small molecules with their mechanism of action. An estimated biological activity profile of drug-like molecules was obtained as an output by using the structural formula of each compound as input. PASS online predicts the biological role of compounds and the potential therapeutic effects of active compounds combined with their chemical entities, which can serve as the basis for bioactivity prediction. The bioactivity and therapeutic effects on neuroprotection were determined based on probability scores, such as the probability of being active (Pa) or inactive (Pi) depending on the structure–activity relationship between the analyzed compounds and their associated parameters (Filimonov et al., 2014). The Molinspiration property explorer web tool was utilized to determine the bioactivities of our screened molecules with respect to predicted bioactivity scores for the six most important properties (Molinspiration, 2025).

## 3 Results and discussion

### 3.1 Ligand-based virtual screening

**FIGURE 2:**
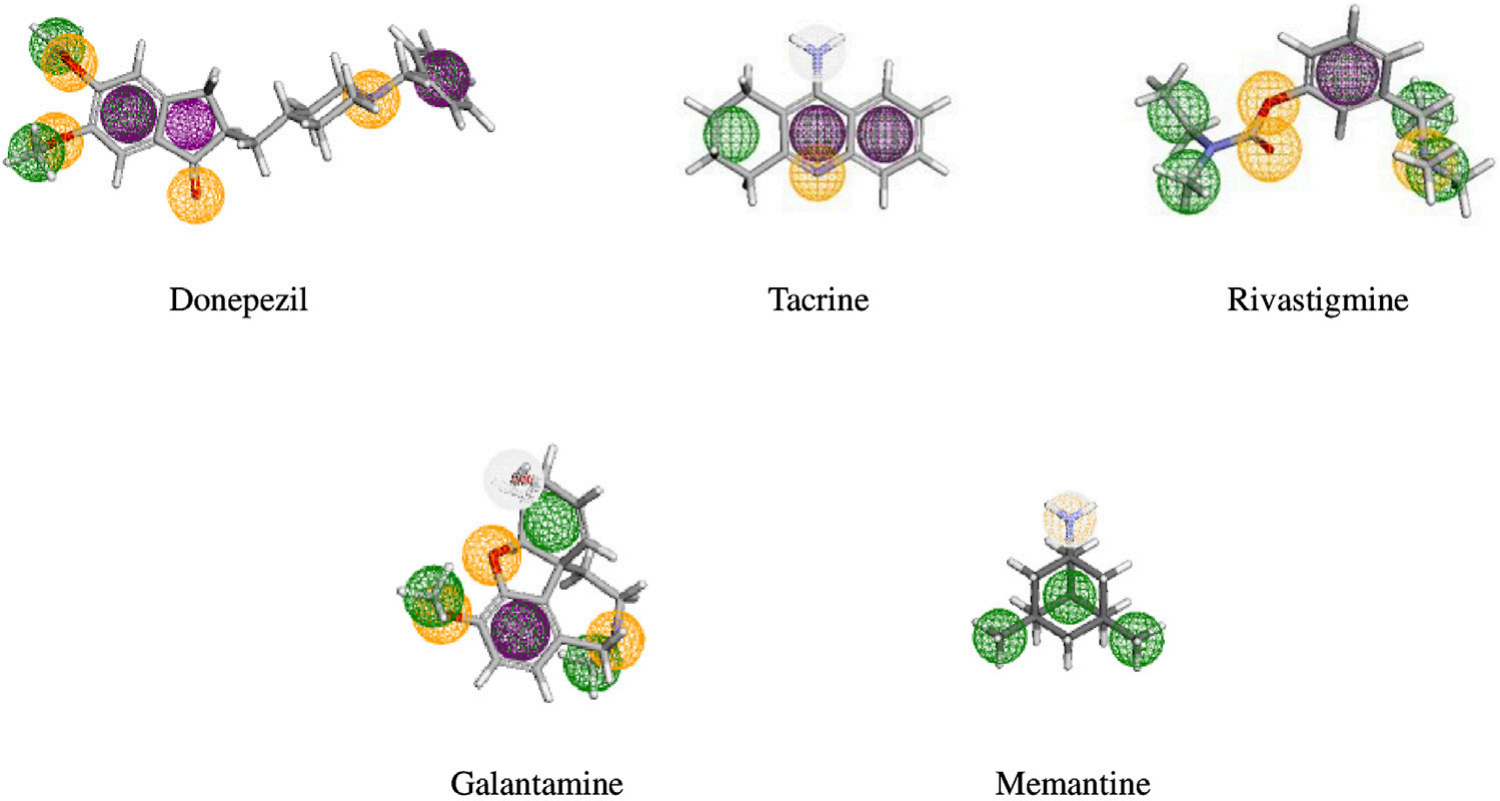
Pharmacophore model of five FDA-approved drugs (donepezil, tacrine, rivastigmine, galantamine, and memantine) used for the treatment of NDDs. The pharmacological features are coded with different colors: aromatic features as a violet ring, hydrophobic feature in a green ring, yellow ring represents the hydrogen bond acceptor, and white represents hydrogen bond donors.

**FIGURE 3:**
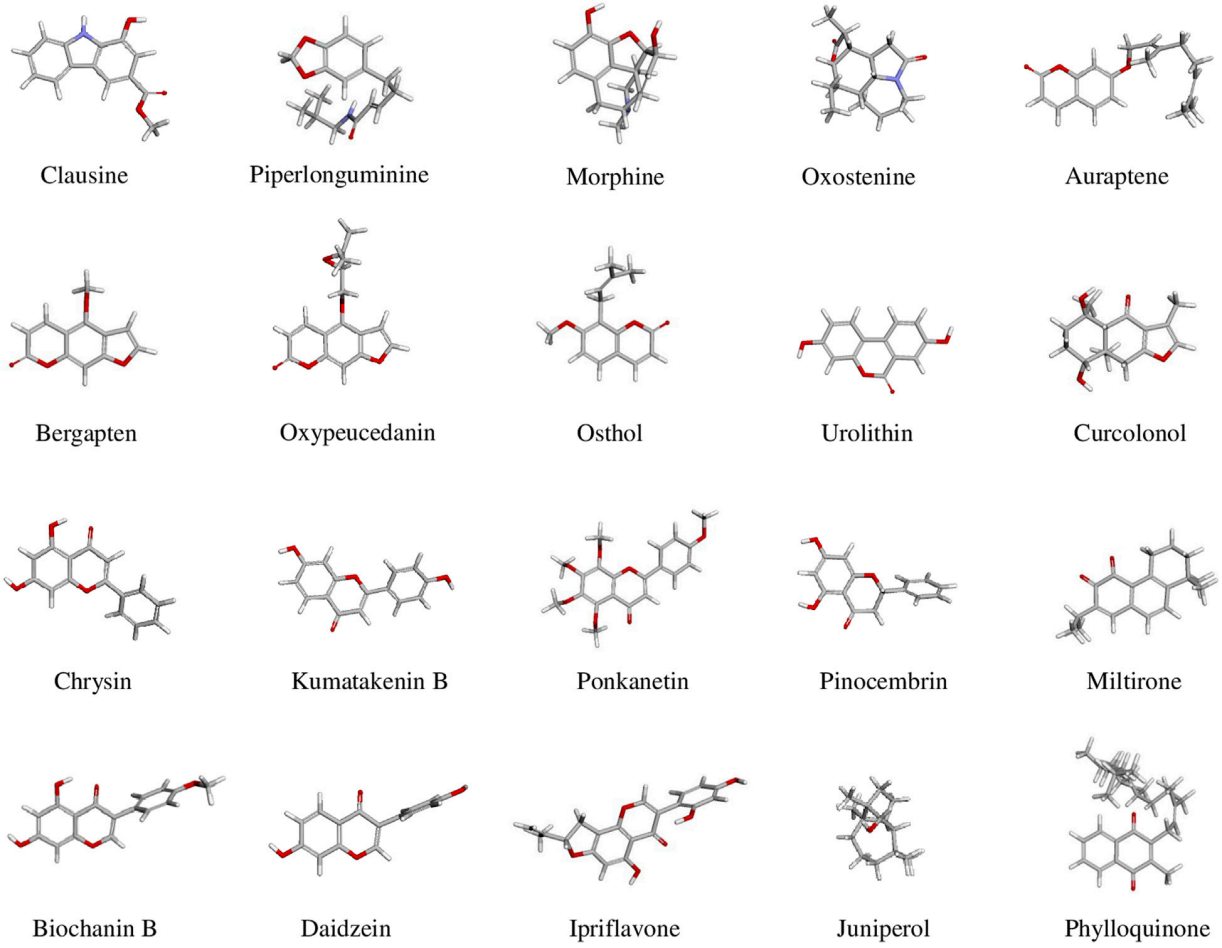
3D conformation of selected BBB-permeable, CNS-active molecules identified in this study through high-throughput virtual screening based on structural similarity. These compounds represent diverse structural classes, including alkaloids, coumarins, flavonoids, and terpenoids. The molecular representations highlight their structural diversity and potential pharmacophores for targeting various neurological diseases.

High-throughput virtual screening used pharmacophore models of five FDA-approved drugs (Figure 2) were used to identify structurally similar active compounds and analogs from Pharmit, ChemMine, and Swiss Similarity, utilizing their integrated databases. To uncover similar structures from natural sources, we initially screened 2,127 compounds with a Tanimoto similarity score of over 0.5, which represented a generally accepted threshold to determine the similarity of bioactive molecules (Szilágyi et al., 2021). The compounds that exhibited extreme similarity to query ligands were screened in order to maintain the uniqueness of the compounds (Figure 3). The molecules were then identified as derivatives of flavonoids, alkaloids, coumarins, and terpenoids (Table 1). Finally, we created a library of bioactive molecules structurally similar to query ligands for further evaluation.

**TABLE 1:** Natural-based small molecules with representative BBB-permeability, CNS activity, mechanism of action, and disease relevance.

Natural product classes	Molecules	BBB permeability CNS activity	Mechanism of action	Disease relevance	References
Alkaloids	Clausine	BBB+ −0.68069 CNS active	Kinase inhibitor GABA C receptor antagonist NF-E2-related factor 2 stimulant	Cerebral ischemia Prion diseases Dementia	Liu et al. (2019)
	Dihydropiperlonguminine	BBB+ −0.22983 CNS active	GABA aminotransferase inhibitor TNF expression inhibitor Neurotrophic factor enhancer Nootropic	Parkinson Disease Cerebral ischemia	Bi et al. (2015)
	Morphine	BBB+ −0.15884 CNS active	Anti-inflammatory IL6 antagonist Nootropic BChE and MAO inhibitor NMDA receptor antagonist	Dementia Parkinson’s disease	Wang et al. (2018)
Coumarins	Auraptene	BBB+ −0.13918 CNS active	Anti-inflammatory, inhibits TNF Antioxidant-free radical scavenger Neurotrophic factor enhancer Kinase and MAO-inhibitor	Dementia Neurodegenerative diseases	Igase et al. (2018)
	Bergapten	BBB+ −0.23549 CNS active	GABA aminotransferase inhibitor Anti-inflammatory, inhibits ILs Antioxidant: NRF2 stimulant MAO B and kinase inhibitor Amyloid-β protein antagonist	Multiple sclerosis Parkinson’s disease Dementia	Salem et al. (2021)
	Oxypeucedanin	BBB+ −0.09974 CNS active	GABA aminotransferase inhibitor Anti-inflammatory Antioxidant: NRF2 stimulant Amyloid-β protein antagonist Cytokine release inhibitor Microtubule stabilization	Multiple sclerosis Parkinson’s disease Dementia	Jalilian et al. (2022)
Flavonoids	Chrysin	BBB+ −0.75733 CNS active	Kinase inhibitor Antioxidant: NRF2 stimulant Nootropic Anti-inflammatory-IL6, TNF antagonist	Dementia Cerebral ischemia	Talebi et al. (2021)
	Kumatakenin B	BBB+ −0.54139 CNS active	Kinase inhibitor Antioxidant: NRF2 stimulant GABA antagonist Anti-inflammatory TNF inhibitor DOPA decarboxylase inhibitor MAO inhibitor Nootropic	Parkinson’s disease Cerebral ischemia Dementia Prion diseases	Li et al. (2025)
	Ponkanetin	BBB+ −0.45751 CNS active	Kinase inhibitor Antioxidant: free radical scavenger Anti-inflammatory IL 6 antagonist	Multiple sclerosis Dementia	Braidy et al. (2017)
Isoflavonoids	Biochanin B	BBB+ −0.31576 CNS active	MAO A and B inhibitor Kinase inhibitor DOPA decarboxylase inhibitor Antioxidant: NRF2 stimulant Anti-inflammatory: IL, TNF antagonist GABA aminotransferase inhibitor Amyloid-β protein antagonist	Dementia Prion diseases Huntington’s disease Amyotrophic lateral sclerosis	Kumar et al. (2024b)
	Daidzein	BBB+ −0.18126 CNS active	DOPA decarboxylase inhibitor Kinase inhibitor MAO A and B inhibitor Antioxidant-NRF2 stimulant Anti-inflammatory: IL6, IL1β, and TNF Amyloid-β protein antagonist	Dementia Prion disease Amyotrophic lateral sclerosis Parkinson’s disease Huntington’s disease	Singh (2025)
	Ipriflavone	BBB+ −0.10076 CNS active	MAO A and B inhibitor Kinase inhibitor NF-E2-related factor 2 stimulant Anti-inflammatory TNF inhibitor DOPA decarboxylase inhibitor Amyloid-β protein antagonist	Dementia Neurodegenerative diseases	Hussien et al. (2021)
Terpenoids	Juniperol	BBB+ 0.4289 CNS active	NF-E2-related factor 2 stimulant Antiinflammatory, inhibits TNF GABA aminotransferase inhibitor	Dementia Parkinson’s disease	Bais et al. (2015)
	Miltirone	BBB+ 0.14967 CNS active	NF-E2-related factor 2 stimulant Anti-inflammatory Kinase inhibitor	Dementia treatment Cerebral ischemia	Feng and Xi (2022)
	Phylloquinone	BBB+ 0.37507 CNS active	Antioxidant: free radical scavenger Anti-inflammatory TNF inhibitor Nootropic	Huntington’s disease Amyotrophic lateral sclerosis	Emekli-Alturfan and Alturfan (2022)

This table highlights a representative subset of the molecules; a full listing can be provided as supplementary material.

### 3.2 Molecular descriptors

Molecular descriptors related to BBB permeability and CNS activity were calculated using the ChemDes web platform, capturing key physicochemical and topological properties of the compounds. A total of 12 important molecular descriptors of active molecules were computed for the analysis, which is relevant to the BBB permeability and CNS activity of molecules. The computed 3D molecular descriptors that include intramolecular bonding (hydrogen bonding), volume (size), polar surface area (topological), surface accessible to the solvent (acidity/basicity), gyration radius, molecular weight, total atom counts, type of angles, number of aromatic rings, number of rotatable bonds, sum of oxygen and nitrogen atoms in the molecule, dihedrals, and molar refractivity were calculated for their predictive ability (Russo et al., 2018).

### 3.3 BBB permeability

**FIGURE 4:**
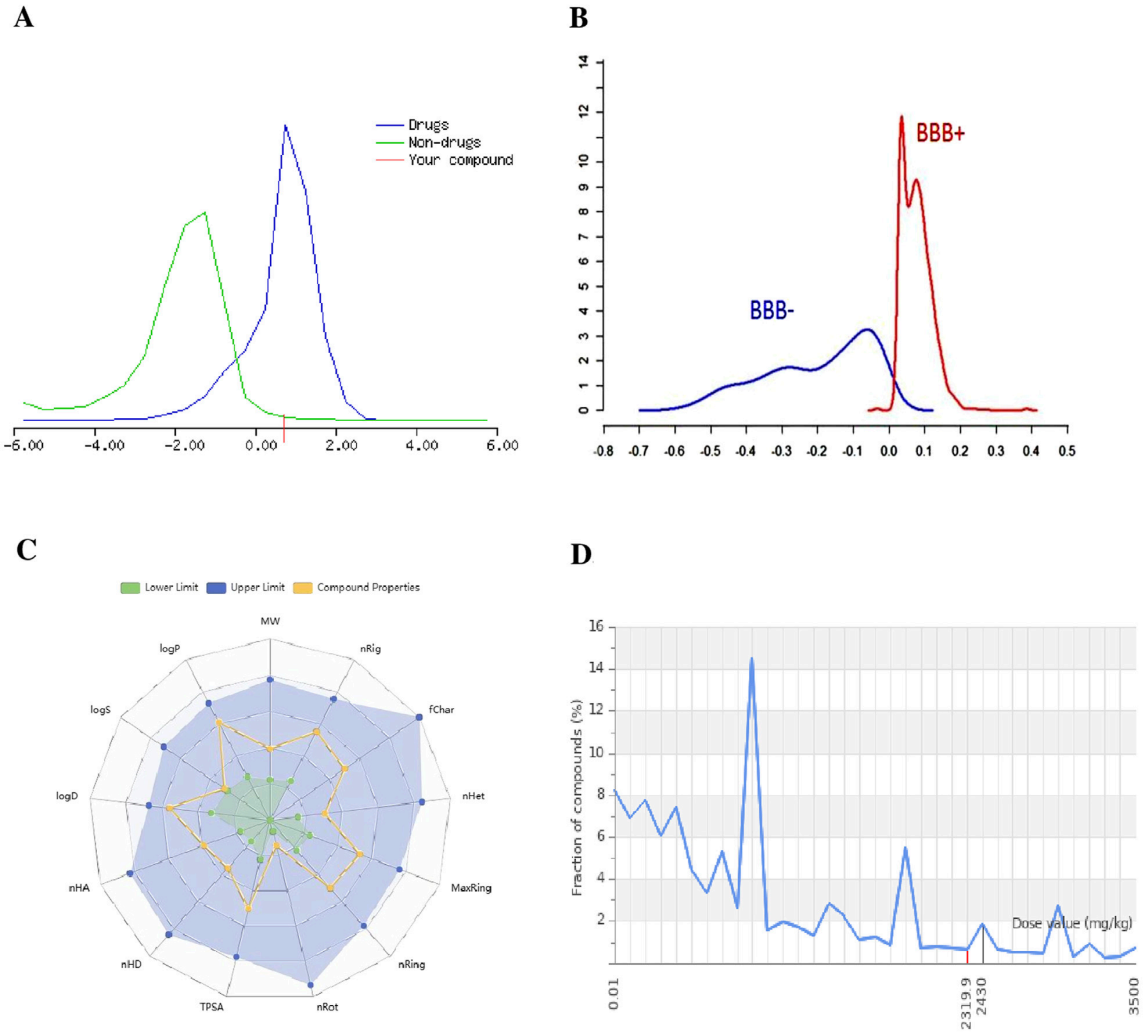
**(A)** Drug-likeness score of the compound (red line) is compared against distributions of known drugs (blue curve) and non-drugs (green curve), indicating its potential as a drug candidate. **(B)** BBB permeability: BBB score predicts the small molecules’ ability to cross the BBB, with “BBB+” indicating permeability and “BBB−” indicating non-permeability. **(C)** Radar plot visualizing key ADME parameters (logP, MW, TPSA, etc.), comparing the small molecule properties (yellow line) to acceptable ranges (green and blue shaded areas). **(D)** Toxicity: predicted LD50 and toxicity class of small molecules.

The BBB permeability of small molecules was evaluated in well-known *in silico*, machine learning, and deep learning models such as BBBper, LightBBB, and Deep-B3. For the BBB model, out of 2,127 molecules, 582 active molecules were identified as BBB-permeable (BBB+), and 1,545 were identified as non-BBB-permeable (BBB−). According to the BBB online predictor, molecules with a threshold BBB−/BBB + score ≥0.02 are considered capable of crossing the BBB, while those with scores below this threshold are classified as non-BBB-permeable (Figure 4B). The identification of these small molecules is essential for drug development, particularly for treatments targeting central nervous system disorders with effective delivery across the BBB. These BBB predictor models achieved an accuracy of above 85% with the best qualitative model.

### 3.4 CNS activity

CNS permeation activity was estimated only for the 582 BBB + small molecules in LogBB_Pred based on their logBB threshold values. BBB permeable molecules were found to have logBB ≥ −1 and were identified as CNS-active (CNS+) molecules (Table 1). CNS MPO scores were obtained for our 582 BBB + molecules based on the desired value of six common physicochemical and pharmacokinetic properties: molecular weight (MW), topological polar surface area (TPSA), number of hydrogen bond donors (HBD), lipophilicity or calculated partition coefficient (clogP), calculated distribution coefficient (clogD), and negative base-10 logarithm of the acid dissociation constant (pKa) of the most basic center of the molecule. The potential small molecules with a generated desirability score value ≥4.0 tend to be identified as CNS-active (Gupta et al., 2024). The identified CNS-active small molecules are highly distributed in the brain and have favorable properties to exert effect within the CNS.

### 3.5 Medicinal chemistry friendliness

The medicinal chemistry filter was applied to small molecules obtained after the BBB-permeable and CNS-active screening. A total of 582 bioactive compounds were subjected to MedChem rule-based filters like PAINS, Brenk alerts, lead likeness, and synthetic accessibility. The analyzed results revealed that most of the compounds were predicted to be PAINS with less alerts, indicating non-interference in biological assays. Brenk filter with less structural alerts indicated non-problematic compounds. Lead-likeness with less violation met the desired criteria, suitable for the optimization of the pharmacodynamic properties of our screened small molecules. Synthetic accessibility score values ranging from 1 (results easy to synthesize) to 10 (results difficult to synthesize), were calculated, with molecules having average score value less than 4 and close to 1 perhaps being relatively easy to synthesize in the laboratory (Bung et al., 2022).

### 3.6 Oral bioavailability

Oral bioavailability incorporates important factors such as solubility (LogS ≤ 6), permeability, and metabolic stability, allowing us to generate a comprehensive bioavailability profile for each molecule. Our analysis revealed that BBB permeable molecules were predicted to have favorable oral bioavailability with good accuracy, depending on the two cutoff threshold values of F ≥ 20% and F ≥ 50% (high oral bioavailability). Notably, we identified high-priority drug-like candidates with predicted human oral bioavailability values F ≥ 20%, suggesting good absorption potential in the gastrointestinal tract and subsequent brain uptake. According to SwissADME, small molecules with a potential bioavailability score of 0.5 were selected during the early stages of the drug discovery process by efficiently identifying promising candidates for oral administration.

### 3.7 ADME

The ADME properties of small molecules were evaluated using online cheminformatics tools such as ADMET lab 2.0 (Figure 4C), Swiss ADME, and pkCSM. Absorption is an important parameter for checking the efficacy of small molecules. All molecules were shown to have Caco-2 and MDCK cell permeability in positive test values with more than 90% intestinal absorption, which is ideal for good absorption. Likewise, all small molecules had a volume of distribution in the acceptable range of 0.04–20 L/kg, indicating uniform drug distribution in the body fluid and its uptake amount in tissues. In the case of metabolism, approximately two-thirds of small molecules were attributed to the human cytochrome P450 family, which includes five liver isozymes—CYP2D6, CYP3A4, CYP1A2, CYP2C19, and CYP2C9—which are responsible for metabolic activity. Additionally, small molecules were reported as potential substrates or non-inhibitors for most cytochrome P450 isoforms. The predicted half-life for the majority of small molecules was longer at >3 h. All screened small molecules were predicted to have favorable excretion, with results from moderate to high clearance rates of 5–15 mL/min/kg; suggesting efficient elimination through the kidneys.

### 3.8 Toxicity profiling

The various types of toxicity factors of active molecules were predicted using ProTox 3.0 online software. All compounds were shown to be highly inactive and less active for organ toxicity, other toxicity endpoints, and Tox21-toxicological pathways. All compounds were predicted with toxicity classes of 4, 5, and 6 (non-toxic) for acute oral toxicity, with lethal dose (LD50) values in the range of 2000 > LD50 > 5,000 mg/kg (Figure 4D), with a prediction accuracy of above 70%. The screened bioactive compounds were predicted to be inactive for organ toxicity, particularly for hepatotoxicity and neurotoxicity. The bioactive compounds were also classified as inactive for the main type of toxicological endpoint. In addition, most of the bioactive compounds were reported to be inactive/less active for toxicological pathways and molecular initiating events, with less probability of binding to toxicity targets. All models are based on a machine learning approach, and results are predicted with a confidence score of above 0.7.

### 3.9 Drug-likeness

The drug-like properties of screened molecules were obtained through Drulito. The evaluated drug-like soft filter that resulted in our potential small molecules meeting all the parameters of the Lipinski “rule of 5” were MW ≤ 500 g mol-1, number of hydrogen-bound acceptors (HBA ≤ 10), hydrogen-bound donors (HBD ≤ 5), and lipophilicity (the partition coefficient between n-octanol and water, CLogP ≤ 5). Ghose and Egan’s rule criteria like MW 160–480, WLog P value between −0.3 and +5, molar refractivity (MR, 40–130) and atom count ≤70, and Veber rule are rotatable bonds (n-ROTB should be ≤10, total polar surface area (TPSA ≤ 140 Å), logarithmic distribution coefficient (ClogD) at physiological pH 7.4 in the range of 1–4 (Stéen et al., 2022; Tamaian et al., 2023). Small molecules that violated more than one of these sets of rules were excluded. The Molsoft tool provided a probability of drug score value around 0–4 (Figure 4A), indicating that the small molecules are considered drug-like molecules.

### 3.10 Side effect resource

The side effects (ADRs, adverse drug reactions) and indications for our list of drug-like molecules were predicted independently based on the structured data obtained from the SIDER database. A total of 582 BBB-permeable small molecules were mapped against known drug side effect pairs related to the CNS. Small molecules in the SIDER4.1 dataset with side effects, resources, and indications were excluded for subsequent validation. Consequently, we screened and filtered out molecules that are reported to have side effects relevant to the CNS, such as anxiety, depression, dizziness, schizophrenia, neurological impairment, and other neurological side effects. In the SIDER dataset, the majority of our active molecules were reported to have very rare (<0.1%) side effects with zero frequency. Additionally, drug indications were described to be 0%, which means that small molecules are not associated with ADR within this resource. Notably, our predictions indicate very rare side effects, highlighting the utility of SIDER in identifying potential safety concerns.

### 3.11 Biological activity and mechanism of action

The biological activities related to neuroprotectivity were evaluated through the PASS online server. PASS online prediction provided the probability of active (Pa) and inactive (Pi) scores for a variety of biological activities, mechanisms of action, and disease relevance for each molecule (Table 1) (Filimonov et al., 2014). All BBB-permeable bioactive molecules were shown to have a higher probability value (Pa > Pi), with high antioxidant, anti-inflammation, and anticholinergic activity. The important key findings relevant to neuroprotective activities for our screened BBB-permeable small molecules are provided in Table 2, which lists bioactivities with high Pa > 0.3. According to PASS prediction, all compounds were found to be nootropic, brain-derived neurotrophic factor enhancers, neuroinflammatory modulators, and have anti-Alzheimer’s and anti-Parkinson’s activity (Filimonov et al., 2014). Molinspiration explored the predicted bioactivity score >0.05 of small molecules that exhibited moderate inhibition of disease-specific kinases, proteases, and enzymes.

**TABLE 2:** Neuroprotective properties and the biological activities of BBB-permeable small molecules relevant to neuronal activity.

Properties	Bioactivity (Pa > Pi)		References
	Stimulant/agonist ↑	Inhibitor/antagonist ↓	
Antioxidant defense	NRF2 stimulant	MAO inhibitor	Filimonov et al. (2014), Kocsis et al. (2025)
Neuro-inflammatory modulators	Anti-inflammatory IL10, 11, and 13 agonist	Cytokine release inhibitor IL6, IL1B, TNF, and IFN antagonist	Filimonov et al. (2014), Kocsis et al. (2025)
Cholinergic activity	Acetylcholine release stimulant Acetylcholine agonist	BCHE, ACHE inhibitor Choline kinase inhibitor	Filimonov et al. (2014), Kocsis et al. (2025)
Neuronal activity	Neurotrophic factor enhancer Nootropic Microtubule stabilization	Aβ aggregation inhibitor Amyloid-β protein antagonist Tubulin antagonist	Filimonov et al. (2014), Kocsis et al. (2025)
Neuronal transmission	Neurotransmitter agonist	NMDA receptor antagonist	Filimonov et al. (2014), Kocsis et al. (2025)

## 4 Discussion

The blood–brain barrier (BBB) has become an important clinical feature in the discovery of small molecules that target neurodegenerative conditions (Sweeney et al., 2019). Effective delivery of neurotherapeutic agents, drugs, and small molecules across the BBB in order to reach the brain is a primary limiting factor for the successful therapy of neurological conditions. During the early stages of drug research, checking for BBB permeation offers crucial information for choosing the appropriate molecules. However, few studies have reported that many compounds failed due to their inability to permeate the BBB rather than a lack of potency to reach the brain, which rendered the BBB a significant obstacle in discovering central nervous system (CNS) drugs (Cornelissen et al., 2023). Therefore, our work aimed to address this challenge by employing *in silico* tools and existing machine learning model-based approaches to screen for BBB-permeable neurotherapeutics (Bicker et al., 2014), CNS-active drug-like small molecules that could serve as potential therapeutic interventions that can effectively target the underlying mechanisms of neurodegenerative diseases (NDDs) such as Alzheimer’s disease, Parkinson’s disease, and amyotrophic lateral sclerosis.

A total of 2,127 molecules were screened using a pharmacological model of five FDA-approved drugs. Ligand-based drug discovery requires a target-site-specific drug in order to understand the chemical space and create novel drug-like molecules (Bung et al., 2022). Hence, we performed high-throughput virtual screening based on structural similarity with respect to the selected list of FDA-approved drugs for NDDs, such as Donepezil, tacrine, memantine, galantamine, and rivastigmine. These pharmacotherapies appear to partially alleviate various clinical symptoms associated with NDDs. However, the therapeutic benefits are often minimal, transient, and non-selective, and are frequently accompanied by adverse side effects (Duc Nguyen, 2023; Chopade et al., 2023). Due to these drawbacks, innovative therapies from natural products and dietary sources are being considered for NDDs. A wide range of molecular descriptors explores trends with CNS active drugs. The involvement of 12 types of molecular descriptors in our study proved to be associated with BBB permeability of existing studies (Jiang et al., 2016; Faramarz et al., 2022) and provided a comprehensive understanding of how these features correlate with drug-like properties. Specific physicochemical descriptors like MW, lipophilicity, TPSA, and HB can influence BBB permeability (log BB) and are positively correlated to brain uptake (Faramarz et al., 2022; Vilar et al., 2010). The druggability of each molecule must adhere to a defined set of standard rules like RO5 to favor BBB permeation and reach the brain via passive diffusion (Pajouhesh and Lenz, 2005). Therefore, molecules that do not obey these criteria are unsuitable for clinical use and are susceptible to various challenges regarding their properties associated with ADME.

BBB-permeable molecules can have varying effects on the BBB structure. Conversely, certain molecules have been shown to restore or maintain BBB integrity by upregulating tight junction proteins and inhibiting inflammatory mediators. Compounds, such as those derived from natural products and dietary sources, have demonstrated both high BBB permeability and neuroprotective effects, with minimal toxicity to the BBB (Sánchez-Martínez et al., 2022a; Sánchez-Martínez et al., 2022b). According to the BBB model of Shaker et al. (2021), and Tang et al. (2022), 582 small molecules were identified as BBB+ with over ∼85% accuracy in differentiating BBB+ and BBB− based on the brain-to-blood ratio (Zhang et al., 2017). BBB-permeable molecules have been found with direct CNS activities based on predicted logBB values, which is crucial for therapeutic efficacy (Garg et al., 2008). A total of six key descriptors were taken into account for CNS multi-parameter optimization. The CNS MPO showed that 75% of the FDA-approved drugs for CNS disorders have high desirability scores ≥4 for testing molecules, correlating well with our screened molecules for better translation into the clinics (Wager et al., 2016). Medicinal chemists and neuroscientists in recent years optimized the identified molecules to obey essential criteria of synthetic accessibility and lead-likeness in order to improve pharmacokinetics and pharmacodynamics, has been reported to enhance brain uptake via BBB (Anthony et al., 2021; Sanghai et al., 2024).

Physicochemical parameters and structural properties of small molecules determine the BBB permeability, particularly in relation to passive diffusion mechanisms (Cornelissen et al., 2023). We found that the oral bioavailability of small molecules with predicted values exceeding the threshold of 30% indicates good absorption potential in the gastrointestinal tract, which is particularly important for brain targeting. Lipophilicity and molecular weight were found to be pivotal in determining bioavailability, aligned with ADME parameters: increased passive permeability, minimal P-gp liability, adequate metabolic stability, long half-lives, and efficient clearance rates (Stéen et al., 2022). In compliance with the existing study, it was reported that an absorption of drugs more than 90% of the administered dose leads to increased BBB permeability. According to ProTox (Banerjee et al., 2024), a virtual lab assessed the safety profile of desired molecules and found them to be non-toxic. In addition, it is reported to be inactive/less active against organ toxicity, toxicity endpoints, and toxicological pathways (Tox21). Adverse drug reactions were assessed in order to avoid unwanted CNS side effects at the site of action (Adenot and Lahana, 2004). The drug-likeness determines whether the druggability of each small molecule that evolved from natural products is BBB-permeable due to lipid solubility, has a high degree of hydrogen bonding, positively charges molecules, a low Mw of 400–600 Da, and the highest TPSA value (Aldewachi et al., 2021; Russo et al., 2018; Cornelissen et al., 2023). Stéen et al. (2022) suggested that the hydrogen bond interactions between a molecule and the hydrophilic portion of the BBB through lipophilicity enable brain uptake.

Neuronal repair processes involve axonal regeneration, synaptic reorganization, and neuroprotection. Molecules that promote these processes are of great interest for treating NDDs and CNS injuries with neuroregenerative potential. Notably, certain flavonoids and terpenoids have been reported to support neuronal repair and neuroprotection. Neuronal repair depends on the neurobiological activity of small molecules based on their bioavailability in the brain (Wang et al., 2022). Emerging evidence indicates that small molecules possess multimodal biological activities, including antioxidant, anti-inflammatory, anticholinergic, and kinase-inhibitory effects. This multifunctionality is increasingly recognized as a significant characteristic of small molecules, enabling them to interact with multiple biological targets and pathways. Activity spectra predict nearly 4000 types of bioactivities based on the structure–activity relationship (Filimonov et al., 2014). Moreover, our findings indicate that small molecules permeating the BBB possess various neuroprotective activities, such as nootropic, BDNF enhancer, and the modulation of neuroinflammatory pathways. These findings align with current therapeutic needs in treating neurodegenerative diseases. However, the existing scientific research provides extensive lists and classes of such compounds, and many have been confirmed to cross the BBB and exert neuroprotective effects (Mohd Sairazi and Sirajudeen, 2020).

The 112 active CNS molecules identified in this study comprise a diverse array of naturally based small molecules, each with demonstrated BBB permeability. Recent research highlights the distinct mechanisms of action across key brain cell types, such as neurons, astrocytes, microglia, and endothelial cells, for effective therapeutic intervention in NDD. Active molecules that reach the brain can regulate CNS homeostasis, reduce neuroinflammation and excitotoxicity, promote neuroprotection by reducing oxidative stress, inhibit apoptosis, and support synaptic plasticity, thus directly modulating neurotransmitter systems, including dopaminergic, glutamatergic, and cholinergic pathways, or promoting neuronal survival, making them relevant for treating conditions such as Parkinson’s disease, Alzheimer’s disease, and amyotrophic lateral sclerosis (Cornelissen et al., 2023; Kocsis et al., 2025; Grabska-Kobyłecka et al., 2023).

The chemical diversity of CNS-active, BBB-permeable molecules, including flavonoids, terpenoids, polyphenols, and alkaloids, are well-documented in natural product research as promising scaffolds for CNS drug development (Isabel et al., 2024). The inclusion of naturally derived compounds is consistent with recent reviews that emphasize the BBB-crossing potential of various natural products (Isabel et al., 2024). Our findings are promising and largely support the ability of specific dietary molecules to promote CNS health, improve neuroprotective mechanisms, enhance cognitive function, and mitigate the risk of NDDs. Furthermore, our results align with existing studies that suggest a correlation between dietary intake and neurological outcomes. Many studies have used multiple ligands and/or small molecules in a single delivery system to maximize brain uptake and targeted delivery with improved BBB permeation (Cornelissen et al., 2023; Isabel et al., 2024; Al Gailani et al., 2022). In future, experimental validation needs to be carried out by ensuring their impact on BBB integrity and the neuroprotective properties of small molecules, which can then be formulated as valuable food additives, food supplements, and nutraceuticals that could provide new insights into neurotherapeutics as promising therapeutic interventions for NDDs.

## 5 Conclusion

Neurodegenerative diseases will continue to rise with an aging population. Nevertheless, the rate of advancement in neurotherapeutics is limited by the complexities associated with drug delivery across the blood–brain barrier (BBB). Thus, the integration of *in silico* techniques and machine learning models can provide greater insights to accurately screen and identify biologically active molecules that can effectively pass through the BBB. A total of 112 effective BBB-permeable neurotherapeutic molecules were identified after screening a library of small molecules. Potential small molecules were then configured for pharmacokinetic properties and pharmacodynamic characteristics for recommendation as primary lead molecules. These active molecules were prioritized by considering important factors like structural similarity, ADME profiling, toxicity endpoints, drug-likeness, bioavailability criteria, and side effects. Resources were applied individually for the evaluation of safety profiles of drug-like small molecules to recognize possible health risks. Moreover, bioactivity studies relevant to neuronal activity revealed that many of these molecules possess the capacity to modulate critical pathways involved in neuroprotection and neuroregeneration. Medicinal chemistry friendliness ensured that only those molecules with optimal drug-like characteristics were made for potential therapeutic use, with improved pharmacokinetic properties, expected central nervous system (CNS) activity, and permeation phenomena at the BBB level. While our results are promising, it is significant to note that the translation of *in silico* predictions into successful clinical applications remains challenging. The complexity of the BBB and the multifactorial nature of neurodegenerative diseases (NDDs) necessitate experimental validation of the identified molecules, as well as their ability to restore BBB integrity and provide neuroprotection. Ultimately, the overall research was aimed at the development of novel neurotherapeutics delivered across the BBB with CNS activity. Additionally, our research underscores the potential for dietary interventions as a strategy to promote brain health and mitigate the risk of NDDs like dementia/Alzheimer’s disease, Parkinson’s disease, amyotrophic lateral sclerosis, and multiple sclerosis. Future research should focus on experimental validation by elucidating the underlying mechanisms and optimizing dietary formulations to maximize their beneficial effects on the CNS.

## Data Availability

The original contributions presented in the study are included in the article; further inquiries can be directed to the corresponding author.
